# Editorial

**Published:** 1846-06

**Authors:** 


### THE MEDICAL EXAMINER.

PHILADELPHIA, JUNE, 1846.

### NEW MEDICAL JOURNAL—ALTERATIONS, ETC.

We have received No. 3 of Vol. 1 of the *Southern Journal of Medicine and Pharmacy*, Published at Charleston, S. C., and Edited by J. Lawrence Smith, M. D., and J. D. Sinkler, M. D.

Not having the earlier numbers, we are unable to copy the *Prospectus* of the editors, or say anything of the design of the publication further than may be derived from the number before us. We discover by the title page that it is a *bi-monthly;* and in addition to the usual subjects embraced in a medical journal, it is to include pharmacy as a principal object.

It is a remarkably neat journal, containing about one hundred and twenty pages each number. The original articles, as well as the notices of books and new publications, are well written, and the selections judicious.

The title of the *Illinois Medical and Surgical Journal*, Edited by Professor Blaney, has been changed to the *Illinois and Indiana Medical and Surgical Journal*, and it is henceforth to appear *bi-monthly*, increased in size from sixteen to ninety-four pages, with a full corps of Editors, viz.: James T. Blaney, M. D., Daniel Brainard, M. D., William B. Herrick, M. D., and John Evans, M. D.

The Journal is not only enlarged but improved in every respect, and if the succeeding numbers of the new series shall equal the first, of which we have no doubt, it will well deserve the patronage of its subscribers.

*The Missouri Medical and Surgical Journal* is hereafter to be under the sole editorial charge of Dr. Thomas Barbour. Dr. J. N. McDowell gives notice that he "declines any further connection with the Journal, for the reason that he is engaged in the publication of his long promised work on Surgery and Surgical Anatomy, which he hopes to have before the medical public in the Fall."

---

### NOSTRUM CERTIFIERS.

Under this head, in the last number of the Western Lancet, we have the names of sundry physicians whose signatures appear in certain publications in commendation of various nostrums, and the Editor announces his determination to continue, from time to time, to publish the names of all such as come under his notice. If he will extend his researches into this quarter he will find some such from whom we might expect better things. The vanity of having their names blazoned in the newspapers, or otherwise made common as household words, turns awry many a weak head. Such men attach their names to a pill, or a mixture, or a quack advertisement, that they may be lifted into notice, on the same principle as the silly terrapin in the fable seized a pole, that on the wings of others it might rise above the earth.

---

### OUR EXCHANGES.

We are sorry to find that some of our brother journalists with whom we exchange do not receive the *Examiner* regularly. We regret this exceedingly, as we know by rather painful experience how annoying such irregularities are, and we endeavour to avoid it so far as it is in our power. With all our desire in the matter, however, miscarriages will happen, and it seems to have occurred, almost regularly, we might say, with the *Southern Medical and Surgical Journal.* The publishers of the Examiner, who exclusively attend to every thing pertaining to its *business affairs,* assure us that it is invariably sent, by mail, carefully directed, to all those with whom we exchange. The same care will be taken in future, and we hope with better success.

---

NATIONAL MEDICAL CONVENTION.

We are indebted to our friend of the *Bulletin of Medical Science* for a slip containing an abstract of the proceedings of the Medical Convention, recently assembled in the City of New York, which will be found in our *Record.* The next meeting, we perceive, is to be held in Philadelphia, on the first Wednesday in May, 1847.

